# User Experiences of a Chatbot for Supporting the Self-Management of Peripherally Inserted Central Catheter for Chemotherapy: Mixed Methods Study

**DOI:** 10.2196/81026

**Published:** 2026-02-11

**Authors:** Bokyung Jo, Su Jin Kim, Min Jeong Kim, Nayeon Kim, Ayoung Lee, Hoyoung Kim, Mangyeong Lee, Juhee Cho

**Affiliations:** 1 Department of Clinical Research Design & Evaluation, SAIHST, Sungkyunkwan University, Seoul, Republic of Korea; 2 Department of Digital Health, SAIHST, Sungkyunkwan University, Seoul, Republic of Korea; 3 Center for Clinical Epidemiology, Samsung Medical Center, Seoul, Republic of Korea; 4 Research Institution for Future Medicine, Samsung Medical Center, Seoul, Republic of Korea; 5 Cancer Education Center, Samsung Medical Center, 81 Irwon‐ro, Gangnam, Seoul, 06351, Republic of Korea, 82 2-3410-1448

**Keywords:** chatbot, chemotherapy, peripherally inserted central catheter, self-care, user experience

## Abstract

**Background:**

A peripherally inserted central catheter (PICC) for vesicant or long-term chemotherapy is recommended for safe and sustainable drug delivery. However, maintaining its benefits requires regular and careful self-management. Although medical staff provide education and telephone consultation, proactive support accessible at any time or location remains limited. Therefore, we developed a rule-based chatbot to support PICC self-management.

**Objective:**

This study aimed to evaluate the feasibility of a chatbot designed to support PICC self-management by examining chatbot use rate, usability, and user experience.

**Methods:**

A mixed methods study was conducted from September to December 2022, adhering to the GRAMMS (Good Reporting of a Mixed Methods Study) guideline. Patients with cancer scheduled for PICC insertion and their caregivers were recruited, as PICC care is commonly performed by patients or cohabiting caregivers. All participants provided written informed consent. The chatbot was designed to provide structured responses based on prespecified dialog trees and to recognize users’ intent using natural language processing. It was delivered through KakaoTalk and accessed on participants’ personal mobile phones without requiring a separate app installation. Participants received face-to-face training at enrollment and were asked to voluntarily use the chatbot for 1 month. Baseline and postintervention surveys assessing usability were administered using paper-based questionnaires. Usage logs were collected from a secure researcher dashboard and analyzed for inquiry topics, free-text inputs, and fallback situations. Semistructured interviews were conducted approximately 1 month after the intervention during outpatient visits, with invitations by telephone, to explore participants’ experiences regarding chatbot use. Quantitative data were analyzed descriptively to summarize participant characteristics, chatbot use, and usability outcomes, while qualitative interview data were analyzed using thematic analysis.

**Results:**

A total of 56 participants were included in the final analysis (mean age 55.4 years, SD 13.7; female: n=39, 70%). Among them, 28 (50%) used the chatbot at least once. Chatbot users were younger than nonusers (51.1 vs 59.6 y; *P*=.02). Of the 25 users who agreed to log analysis, 347 inquiries were recorded; frequent topics included catheter care (126 observations), managing daily life (85 observations), symptoms (72 observations), and heparin use (55 observations). Among the 23 users who completed the usability survey, 20 (87%) reported that the chatbot was helpful for PICC-related issues. Qualitative interviews (N=56) identified 3 major benefits—information accessibility, effective guidance, and psychosocial support—while also revealing unmet needs related to conversational issues, user experience issues, and lack of personalization.

**Conclusions:**

A rule-based chatbot designed to support PICC self-management demonstrates potential to enhance information accessibility, provide practical guidance, and offer psychosocial support. However, limitations related to conversational flexibility, interface usability, and personalization highlight the need for future development incorporating large language models. Longitudinal and multisite studies are warranted to assess sustained user engagement and clinical outcomes.

## Introduction

A peripherally inserted central catheter (PICC) is inserted through a deep peripheral vein of the upper arm and threaded into a large vein above the right side of the heart [1]. This medical device is safe and useful for patients with cancer requiring long-term and/or frequent chemotherapy as well as a vesicant regimen with a risk of skin necrosis [2]. However, the nature of long-term administration makes PICC-related complications common in daily living. According to a prospective cohort study with 477 patients with cancer, 81 (17.0%) patients experienced PICC-related complications during the study period, resulting in an incidence rate of 1.59 per 1000 catheter days [3]. A systematic review reported that approximately 25% of central venous access devices were removed or failed before therapy was completed [4]. A meta-analysis of 11 studies reported that PICCs were associated with a 2.5 times higher risk of deep vein thrombosis compared with other central venous catheters [5].

PICC-related complications can be associated with practitioners’ training and competence in PICC insertion as well as patient self-management [6-8]. Inadequate maintenance can lead to a surge in unplanned clinical encounters, including infections, bleeding, skin allergies, pain at the insertion site, and tube blockage [3,9,10]. As outpatient chemotherapy becomes increasingly common in many hospitals, the role of patients and caregivers in PICC care has become more important [11-13]. Thus, empowering patients to manage PICC lines safely after insertion helps mitigate PICC-related complications, improving treatment satisfaction [14,15]. Hospitals provide PICC-related education programs for this purpose, but these are somewhat limited in addressing the ongoing care needs or real-life challenges faced by patients.

Harnessing chatbots may be a solution for these challenges [16-18]. Chatbots simulate human interaction, offering real-time responses and guidance in text, voice, or visual formats [19]. According to a systematic review, use cases for chatbots in oncology are versatile, including screening, treatment planning, informational support related to treatment, remote patient monitoring, and emotional support [20]. A feasibility study with 9 patients with cancer reported that a chatbot was helpful in revealing serious health conditions, such as fever, skin rash, or abnormal sensitivity in the extremities [21]. A randomized controlled trial with 142 patients with breast cancer found that the perceived quality of cancer information provided by a chatbot was statistically noninferior compared with the information provided by physicians [22]. Moreover, nearly 90% of patients with cancer answered that chatbots were helpful in acquiring knowledge related to cancer or tracking treatment progress [23,24].

Although chatbot-based health care has many applications, chatbot feasibility in the context of supporting PICC self-management remains unclear [10,22,25]. The general public is unfamiliar with chatbots in the medical context owing to the relative newness of the technology compared with other digital solutions. This is particularly true for older adults, who form the majority of the population of patients with cancer. Therefore, the aim of this study was to investigate the feasibility of a chatbot for PICC self-management and determine users’ characteristics, usage patterns, and satisfaction.

## Methods

### Design and Participants

A mixed methods study was conducted from September to December 2022, adhering to the GRAMMS (Good Reporting of a Mixed Methods Study) guideline. Quantitative analysis alone could identify who used or did not use the chatbot but could not explain the underlying reasons for these behaviors. To understand the contextual factors influencing adoption and engagement, qualitative inquiry was incorporated. Quantitative surveys and user logs provided objective data on participant characteristics, chatbot use, and usability, whereas qualitative interviews explored reasons for use or nonuse, perceived benefits, and barriers to use. This study used a sequential mixed methods design, in which quantitative surveys were conducted first, followed by qualitative interviews.

Patients with cancer and their caregivers were recruited from the Cancer Education Center of Samsung Medical Center in Seoul, South Korea. All patients scheduled to undergo PICC insertion were required to attend a face-to-face education session prior to the procedure. We consecutively contacted and recruited potential participants following each education session. As some patients with a PICC line were not scheduled to receive chemotherapy, an oncology nurse screened eligible patients and their caregivers prior to recruitment. We used convenience sampling, and individuals who voluntarily agreed to participate were enrolled in the study. Those who were 19 years or older, scheduled for PICC insertion (patients only), owned a smartphone, and had ever used KakaoTalk (most popular instant message service in Korea) were eligible for the study. However, those with any cognitive or physical problems with using a smartphone and who were scheduled for PICC line removal within a month were excluded. Following the provision of written informed consent, participants received approximately 15‐20 minutes of training on how to access and use the chatbot. The session covered the basic user interface, methods for interacting with the chatbot, troubleshooting technical issues, and general precautions for use. Participants practiced using the chatbot under the guidance of research staff.

### Chatbot Description

**Figure 1.**
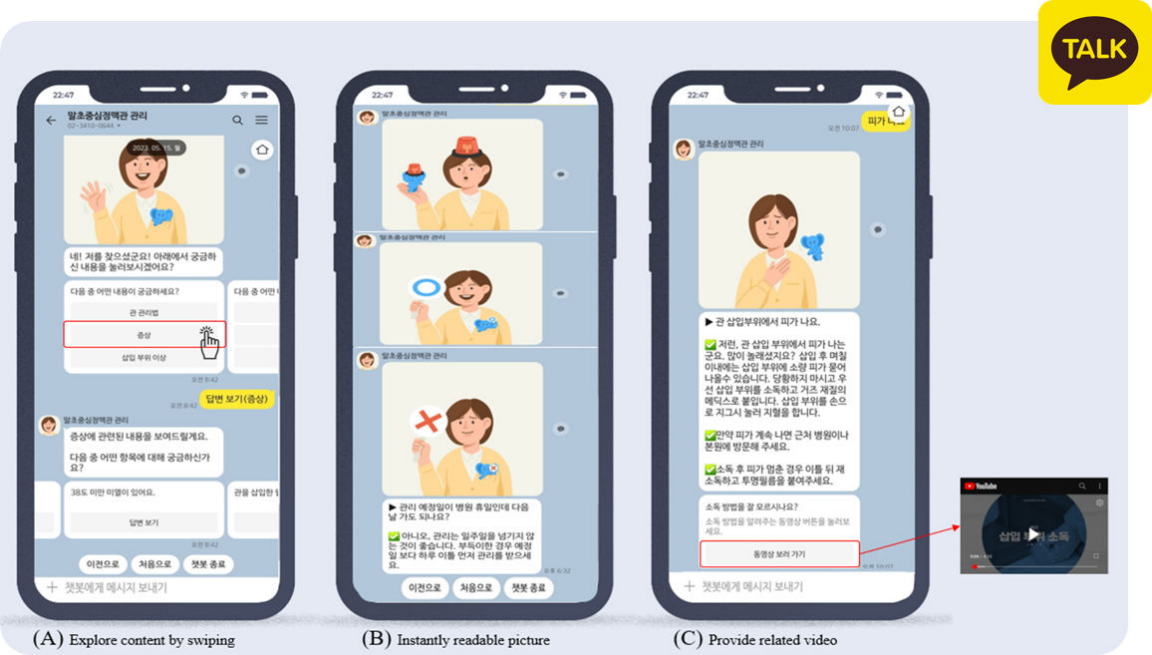
Examples of the peripherally inserted central catheter (PICC) chatbot.

Participants were asked to voluntarily use a chatbot prototype to support PICC management for 1 month. The chatbot was a rule-based system that provided structured responses along prespecified dialog trees. It was technically implemented by Skelter Labs Corp (Figure 1) and integrated into KakaoTalk, a popular instant messenger in Korea [26]. The main purpose of the chatbot was to provide structured information regarding PICC management according to user inquiries. In the welcome message, the chatbot told the participants what the chatbot can and cannot do. The chatbot clarified that it has not been designed to replace clinical practice or make clinical decisions.

Users could ask the chatbot questions by tapping buttons listed according to prespecified dialog trees or typing their queries directly. Regarding the users’ free text, the chatbot was enabled to understand the intent based on natural language processing. Dialog trees were constructed according to 6 main themes: catheter management, adverse events with the catheter, coping with emergencies, symptom management, precautions during daily life, and heparin-related inquiries. These themes were determined by reviewing records of PICC consultations in which patients had previously asked questions. The most frequently asked questions for each theme were displayed in a carousel-type list, allowing users to navigate through the available inquiry options with minimal scrolling. The chatbot used the health information about PICC management developed by advanced practice nurses in charge of patient education for PICC management. Considering the role of chatbots as social actors, health information in the chatbot was delivered through “Seulgi” (“wise” in Korean), a virtual character with the appearance of a nurse and a friendly tone. However, some information too complicated to convey through text alone was delivered via photos or videos. In addition, for irrelevant or ambiguous inquiries, the chatbot delivered fallback messages that reminded users of the chatbot’s original purpose and highlighted the contact number of the cancer education center for additional help.

### Measurement

Chatbot feasibility was evaluated using triangulation with quantitative usability survey, user logs, and qualitative interviews. The investigated topics included questions such as who voluntarily used chatbots, why they used them, what the most frequently asked questions were, and how satisfied users were.

#### Quantitative Usability Survey

Regarding the usability survey, the participants were asked to complete a questionnaire at baseline and postintervention. The baseline questionnaire collected sociodemographic information including age, sex, educational level, occupation, income, area of residence, cohabitation status, and comorbidities. The postintervention questionnaire assessed chatbot adoption, reasons for using (or not using) the chatbot, and user satisfaction. Regarding user satisfaction, a questionnaire was specifically developed by referring to existing reliable and valid user experience questionnaires [27]. The questionnaire covered ease of use (n=5), usefulness (n=7), social interaction with the chatbot (n=5), overall satisfaction (n=1), intention to recommend it to others (n=1), and intention of continuous use (n=1). The responses were interpreted on a 5-point Likert scale, with higher scores indicating greater satisfaction with chatbot usage (0=strongly disagree; 1=disagree; 2=neutral; 3=agree; 4=strongly agree).

#### User Logs

In terms of user logs, the collected data pertained to what buttons users tapped and the inquiries they directly typed. All user logs contained anonymized unique identifiers, which did not allow the decryption of their original identifications.

#### Qualitative Interview

To derive additional insights about the chatbot experience, face-to-face or telephone interviews were conducted with those who agreed to participate. The interviews, lasting 30‐60 minutes, were based on a semistructured questionnaire including PICC-related problems during the study period, awareness of the chatbot, additional reasons for using (or not using) the chatbot, perceived benefits (only for users), and unmet needs. In-depth interviews were typically conducted in a quiet counseling room within the Cancer Education Center. For participants who were unable to revisit the hospital, interviews were conducted by telephone. Each interview was carried out by a team of 3 trained interviewers (BJ, ML, NK), including 1 oncology nurse specialist and 2 additional researchers with prior training in qualitative interviewing. During each session, 1 interviewer led the discussion, while another assisted by taking notes and managing the recording process. All interviewers had prior experience in conducting focus interviews and qualitative data collection. Participants were informed that the interviewers were members of the research team. All interviews were audio-recorded and transcribed.

### Data Analyses

Descriptive statistics were used to compare characteristics between chatbot users and nonusers, identify the most frequently asked questions, and determine user satisfaction. Continuous variables were presented as means and SDs and categorical variables as frequencies and percentages. Group differences between users and nonusers were examined using independent *t* tests for continuous variables and chi-square tests for categorical variables. Graphical depictions were presented to show the main reasons for using (or not using) the chatbot. Regarding user satisfaction, all responses were dichotomized into “positive” (agree and strongly agree) and “negative” (all other responses) and summarized in frequencies. All quantitative analyses were explanatory in nature. Statistical tests were 2-sided, and statistical significance was defined using a 95% CI and a *P*<.05. Participants with missing values for a given variable were excluded from the corresponding analysis but retained in other analyses. Because the proportion of missing data was small, missingness was assumed to be missing completely at random, and no imputation was performed. All statistical analyses were performed using R (version 4.1.2; R Foundation for Statistical Computing).

For the qualitative data, we conducted thematic analyses based on Braun and Clarke’s method [28]. Three researchers (BJ, ML, MJK) independently read the transcripts, generated initial codes, and organized them into categories and themes related to participants’ motivations for chatbot use, perceived benefits, unmet needs, and contextual factors of PICC self-management. A shared coding framework and codebook were developed through iterative discussions. Using investigator triangulation, at least 3 researchers coded each transcript independently, compared results, and resolved discrepancies by consensus. The final themes were reviewed jointly to ensure coherence and distinction and by a principal investigator who audited the overall analytic process. Representative quotations were selected to illustrate key themes.

Quantitative and qualitative data were integrated during the interpretation phase using a narrative approach [29]. Qualitative findings contextualized quantitative results by explaining the reasons for chatbot use or nonuse. Integration discussions were conducted by the research team to ensure coherence between datasets.

### Ethical Considerations

The study was reviewed and approved by the Institutional Review Board of the Samsung Medical Center (IRB No. SMC 2022-07-059). Written informed consent was obtained from all participants. All collected data were deidentified, and access was restricted to authorized personnel. Participants received remuneration of 30,000 KRW (approximately US $25) upon completion of the study.

## Results

### Participant Characteristics

During the study, 5‐10 individuals received PICC-related education each day, and 78 individuals initially consented to participate. Among those, 56 who completed the study were included in the final analysis. Among them, 28/56 (50%) used chatbots (Table 1). Chatbot users were more likely to be younger (51.1 y vs 59.6 y; *P*=.02) and female (24/28, 86% vs 15/28, 54%; *P*=.02) compared with nonusers. The chatbot user group tended to have a higher educational level, but there was no statistical significance (19/28, 68% vs 13/28, 46%); *P*=.17). No significant differences were observed between chatbot users and nonusers in terms of the type of participants (patients vs caregivers), working status, family living together, comorbidities, income, and residence area. In the patient subpopulation, chatbot users tended to have breast cancer (13/17, 76% vs 10/20, 50%; *P*=.28), present with early-stage disease (I or II; 11/17, 65% vs 8/20, 40%; *P*=.18), and undergo curative chemotherapy (17/17, 100% vs 17/20, 85%; *P*=.29; Table 2).

**Table 1.** Participant characteristics.

	Overall (N=56)	Users (n=28)	Nonusers (n=28)	*P* value
Age (y), mean (SD)	55.4 (13.7)	51.1 (14.4)	59.6 (11.8)	.02^a^
Sex, n/N (%)				.02^b^
Male	17/56 (30)	4/28 (14.3)	13/28 (46.4)	
Female	39/56 (70)	24/28 (86)	15/28 (54)	
Participant type, n/N (%)				.57^b^
Patients	37/56 (66)	17/28 (61)	20/28 (71)	
Caregivers	19/56 (34)	11/28 (39)	8/28 (29)	
Educational level, n/N (%)				.18^b^
Below high school	24/56 (43)	9/28 (32)	15/28 (54)	
Above college	32/56 (57)	19/28 (68)	13/28 (46)	
Working status (yes), n/N (%)	27/56 (48)	16/28 (57)	11/28 (39)	.28^b^
Family living together (yes), n/N (%)	49/56 (88)	23/28 (82)	26/28 (93)	.42^b^
Chronic disease (yes), n/N (%)	33/56 (59)	18/28 (64)	15/28 (54)	.59^b^
Household monthly income (US $), n/N (%)				.25^b^
<1700	14/55 (25)	5/27 (19)	9/28 (33)	
1700‐4200	26/55 (47)	13/27 (48)	13/28 (46)	
>4200	15/55 (27)	10/27 (37)	5/28 (19)	
Residence area, n/N (%)				.99^b^
Metropolitan	23/55 (42)	11/27 (41)	12/28 (43)	
Nonmetropolitan	32/55 (58)	16/27 (59)	16/28 (57)	

a Independent *t* test was used for continuous variables.

b Chi-square test was used for categorical variables.

**Table 2.** Cancer type and stage characteristics of the patients (n=37).

	Chatbot users (n=17)	Chatbot nonusers(n=20)	*P* value
Age (y), mean (SD)	53.9 (13.7)	61.7 (8.8)	.04^a^
Sex, n/N (%)			.08^b^
Male	1/17 (6)	7/20 (35)	
Female	16/17 (94)	13/20 (65)	
Type of cancer, n/N (%)			.28^b^
Breast	13/17 (76)	10/20 (50)	
Lung	1/17 (6)	3/20 (15)	
Lymphoma	1/17 (6)	5/20 (25)	
Others	2/17 (12)	2/20 (10)	
Stage, n/N (%)			.18^b^
I	3/17 (18)	0/20 (0)	
II	8/17 (47)	8/20 (40)	
III	5/17 (29)	9/20 (45)	
IV	1/17 (6)	3/20 (15)	
Treatment intent, n/N (%)			.29^b^
Curative	17/17 (100)	17/20 (85)	
Palliative	0/17 (0)	3/20 (15)	
Educational level, n/N (%)			.42^b^
Below high school	7/17 (41)	12/20 (60)	
Above college	10/17 (59)	8/20 (40)	
Working status (yes), n/N (%)	14/17 (82)	15/20 (75)	.89^b^
Family living together (yes), n/N (%)	14/17 (82)	19/20 (95)	.48^b^
Chronic disease (yes), n/N (%)	11/17 (65)	9/20 (45)	.39^b^
Household monthly income (US $), n/N (%)			.20^b^
<1700	5/17 (29)	8/19 (42)	
1700‐4200	6/17 (35)	9/19 (47)	
>4200	6/17 (35)	2/19 (11)	
Residence area, n/N (%)			.99^b^
Metropolitan	5/16 (31)	7/20 (35)	
Nonmetropolitan	11/16 (69)	13/20 (65)	

a Independent *t* test was used for continuous variables.

b Chi-square test was used for categorical variables.

### Reasons for Using (or Not Using) a PICC Chatbot

**Figure 2.**
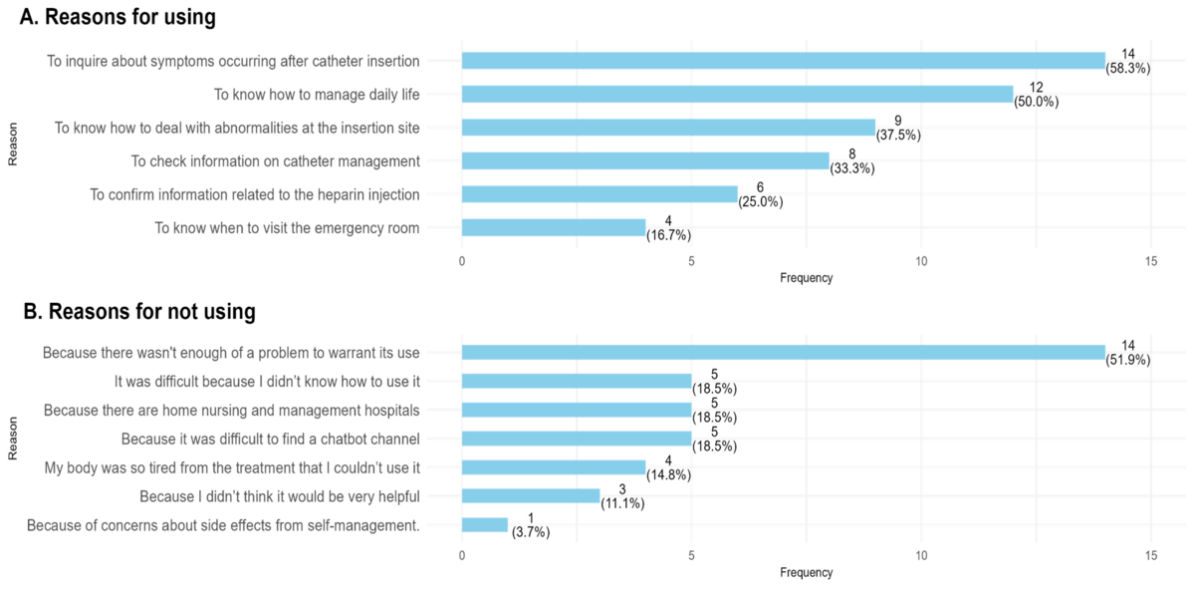
Main reasons for using (or not using) the peripherally inserted central catheter (PICC) chatbot.

Regarding the reasons for using the chatbot, 58.3% (n=14) were for inquiries about symptoms after PICC insertion, followed by learning how to manage daily life (n=12, 50%) and understanding how to deal with abnormalities at the insertion site (n=9, 37.5%; Figure 2). Additional reasons mentioned in the qualitative interviews were repetitive learning to acquire information and the management of anxiety about medical services at local hospitals (Multimedia Appendix 1).

Among the chatbot nonusers, 14/27 (52%) did not have any issues due to their PICC (Figure 2). Additionally, 10 nonusers mentioned that they did not know how to use the PICC (n=5) or that it was difficult to access (n=5). Additional reasons mentioned in the qualitative interviews were a lack of trust in chatbots and low expectations for chatbots (Multimedia Appendix 2).

### Questions Most Frequently Asked of a PICC Chatbot

A total of 25 users agreed to share their logs. During the study period, 347 inquiries were identified and categorized into 4 main themes: catheter care (126 observations), managing daily life (85 observations), symptoms (72 observations), and heparin use (55 observations; Table 3). Additionally, 9 cases were requests to contact the oncology nurse directly (Multimedia Appendix 3).

**Table 3.** User inquiries through the peripherally inserted central catheter (PICC) chatbot (n=25; 347 observations).

Themes and subthemes	observations, n (%)
Within the constructed scope	
Catheter care (126 observations)	
When to change dressing	22 (17)
Buying dressing materials	17 (14)
Intravenous medications through the catheter	15 (12)
When to remove the catheter	13 (10)
Management video	9 (7)
Change of management hospital	9 (7)
Change schedule	8 (6)
PICC part damage	4 (3)
Disinfection supplies	2 (2)
Catheter length abnormality	2 (2)
Managing daily life (85 observations)	
Workouts	24 (28)
Showers	14 (16)
Travel and flying	11 (13)
Driving	11 (13)
Wearing clothes	11 (13)
Publicly used facilities	8 (9)
Cane	2 (2)
Symptoms (72 observations)	
Fever	38 (53)
Bleeding	10 (14)
Pain at insertion site	10 (14)
Pins and needles	5 (7)
Pus	2 (3)
Rash	2 (3)
Swelling	2 (3)
Bruising	1 (1)
Heparin use (55 observations)	
Timing of heparin injection	44 (80)
Dosage	4 (7)
Refill	3 (5)
Storage	2 (4)
Chills and high fever after injection	0 (0)
Using a pump for injecting chemotherapy	0 (0)
Injection not feasible owing to catheter blockage	0 (0)
Outside the constructed scope	
Catheter care (126 observations)	
Blood pressure measurement	9 (7)
Catheter condition abnormality	7 (6)
Dressing problems	3 (2)
How to disinfect	3 (2)
Care after catheter removal	2 (2)
Area suitable for disinfection	1 (1)
Managing daily life (85 observations)	
Painkillers	1 (1)
Manual therapy	1 (1)
Sleep	1 (1)
Hip bath	1 (1)
Symptoms (72 observations)	
Itching	1 (1)
Diarrhea	1 (1)
Heparin use (55 observations)	
Just “heparin”	2 (4)

a Not available.

Regarding catheter care, the most frequently asked question was about the timing for changing dressings (22/126, 17.5%), followed by buying dressing materials (17/126, 13.5%) and taking other intravenous medications through the catheter (15/126, 11.9%). Regarding the management of daily life, workout-related inquiries (24/85, 28%) were the most common, with additional concerns including showers (14/85, 16%), travel (11/85, 13%), driving (11/85, 12.9%), and wearing clothes (11/85, 13%). In terms of symptoms following catheterization, fever (38/72, 53%) was the primary concern, followed by bleeding (10/72, 13.9%) and pain at the insertion site (10/72, 14%). The timing of the heparin injection (44/55, 80%) was the most frequent heparin-related inquiry. During the study period, there were 705 interactions with the chatbot, and we identified a total of 347 user inquiries. Of these, 100 were free-text inputs, and 69 (69%) were successfully recognized by the chatbot. The 31 failed cases occurred because users did not provide sufficient context in their inquiries, entering only single terms, such as “injection,” “not detachable,” or “heparin.” As the fallback scenario was designed with limited functionality, the chatbot could not prompt users to clarify vague inputs.

### Usability Results of the PICC Chatbot

Among the 28 chatbot users, 23/28 (82%) completed the questionnaire, with 22/23 (96%) agreeing that the chatbot was easy to use and 21/23 (91%) mentioning that it was useful for managing their PICC catheter (Table 4). Furthermore, 17/23 (74%) felt like they were conversing with a person during interactions with the chatbot. All participants agreed that the chatbot’s responses were trustworthy, 21/23 (91%) expressed the intention to continue using the chatbot while the catheter was in place, and 22/23 (96%) were satisfied with using the chatbot. All participants (23/23, 100%) recommended the use of chatbots for other patients using catheters.

**Table 4.** Result of the user experience survey (n=23).

Question (out of 80 points)	Mean (SD)	Negative, n (%)	Positive, n (%)
Using the chatbot was challenging (reverse coded).	3.35 (0.98)	1 (4)	22 (96)
I found the explanations provided by the chatbot easy to understand.	3.00 (1.04)	2 (9)	21 (91)
I could promptly address any mistakes made while using the chatbot.	2.96 (0.82)	1 (4)	22 (96)
The text displayed by the chatbot was easy to read.	3.17 (0.72)	0 (0)	23 (100)
I found it easy to locate the information necessary for catheter management using the chatbot.	3.00 (0.74)	0 (0)	23 (100)
The chatbot accurately understood my intentions.	2.65 (0.98)	3 (13)	20 (87)
The chatbot was helpful in addressing issues encountered during catheter management.	2.96 (1.02)	3 (13)	20 (87)
The chatbot responded promptly whenever I had a question.	3.22 (0.74)	0 (0)	23 (100)
The chatbot satisfied my curiosity regarding catheter management.	3.17 (0.89)	1 (4)	22 (96)
I consider chatbots essential for managing my catheter.	3.30 (0.63)	0 (0)	23 (100)
Using a chatbot for catheter management proved beneficial for me.	3.09 (0.95)	2 (9)	21 (91)
The chatbot helped alleviate my anxiety during catheter management.	3.17 (0.89)	1 (4)	22 (96)
The chatbot empathized with me.	2.96 (1.07)	3 (13)	20 (87)
The chatbot responded appropriately to the situation.	2.96 (0.88)	1 (4)	22 (96)
I felt like I was talking to a person through the chatbot.	2.26 (1.14)	6 (26)	17 (74)
The chatbot felt friendly during the conversation.	2.65 (0.98)	3 (13)	20 (87)
The chatbot’s responses were trustworthy.	3.13 (0.69)	0 (0)	23 (100)
I would recommend that other patients with catheters give the chatbot a try.	3.39 (0.72)	0 (0)	23 (100)
I intend to keep using the chatbot for catheter management.	3.22 (1.00)	2 (9)	21 (91)
Overall, I was satisfied with chatbot use for catheter management.	3.09 (0.85)	1 (4)	22 (96)

### Perceived Benefits and Unmet Needs of Using a PICC Chatbot

#### Overview

A total of 56 qualitative interviews were conducted. Regarding the perceived benefits of using the chatbot, 3 themes were identified from chatbot users’ statements. For unmet needs, 3 themes were identified from the statements of chatbot users and nonusers. Detailed representative quotes are provided below, with a more comprehensive overview in Multimedia Appendix 4.

#### Perceived Benefits

In terms of the perceived benefits of using the chatbot, 3 main themes—information accessibility, effective guidance, and psychosocial support—were identified.

##### Information Accessibility

Users found the chatbot useful for accessing PICC-related information anytime they had questions. They felt that the information was easy to understand because it was broken down into small chunks, making it more comprehensible for them.

*It was very helpful because I was able to receive an immediate response in real time. […] And when I needed information, I was able to simply inquire through KakaoTalk.*

[Female, 60s, patient, P56]

*I would like to say that chatbots are understood more clearly and immediately because they provide information in a brief format, unlike paper materials. […] Anyway, chatbots are understood instantly.*

[Female, 40s, caregiver, P06]

##### Effective Guidance

Users agreed that the chatbot was useful for managing PICC-related problems as it provided clear instructions that helped the users take the necessary actions with confidence, thereby enhancing their self-efficacy in improving self-management. Some users described it as a practical guide that bridged knowledge gaps and supported self-management. Beyond self-management support, the chatbot directly connected users to appropriate health care providers when their needs exceeded the system’s capabilities. A user reported that when proper PICC line management was unavailable at a nearby hospital, the chatbot facilitated immediate connection to an oncology nurse from the cancer center where she was being treated, enabling her to receive timely support.

*I checked whether it was correct to attach the tape after it had completely dried. […] I checked this and that and also double checked, “What am I doing wrong?”*

[Female, 60s, caregiver, P38]

*When I looked at this here [chatbot], I thought I could figure out a solution.*

[Male, 70s, Patient, P20]

*After doing some searching on the chatbot, I got in touch with the hospital again. The medical staff gave me a consultation, and I made an appointment for the next day. […] The best part was that it was directly connected to the hospital.*

[Female, 40s, caregiver, P06]

*When I searched, the chatbot suggested that it might be a blood clot. So I quickly received primary treatment at the local hospital’s emergency room and then came back to the main hospital to have the PICC-line removed.*

[Female, 20s, caregiver, P42]

##### Psychosocial Support

Users felt secure while having their PICC line because they knew that the chatbot was always available to them regardless of time and place constraints. Users were easily frightened by uncertainties surrounding their PICC-related concerns. However, access to credible information helped alleviate such fears. One user said that the chatbot helped her prepare coping strategies for potential symptoms in advance, which increased her sense of relief for managing her line. Additionally, users reported feeling reassured when they learned through the chatbot that their symptom experiences were common with other patients.

*If something went wrong, I needed to find a solution quickly, and I thought, “Who should I ask about this?,” but I really relied on this kind of thing. […] I depended on it and asked questions here [chatbot], so I felt much less anxious. Definitely.*

[Female, 70s, patient, P34]

*It really helped me when I looked up things I was curious about, such as when to go to the emergency room, when to switch hospitals, and what to do if my tube falls out. Knowing this information ahead of time made me feel a little more at ease.*

[Female, 60s, caregiver, P19]

*I was looking into whether other people have these symptoms and if this is common or just something I am experiencing.*

[Female, 60s, caregiver, P19]

*I think it helped reduce my anxiety a lot. I realized I had been more scared than I expected, but after getting information from the chatbot, I thought, “I don’t need to worry that much.” For example, learning that catheter disinfection isn’t something to be that afraid of … and also finding out that I can go about daily life and even exercise without any major issues, that was really helpful.*

[Female, 30s, patient, P43]

### Unmet Needs

While users recognized the benefits of the chatbot, several limitations highlighted areas for improvement to enhance user experience and functionality. Through the qualitative analysis, 3 main themes emerged regarding the chatbot’s limitations: conversational issues, user experience issues, and lack of personalization.

#### Conversational Issues

A key challenge reported by users was that the chatbot sometimes failed to provide the appropriate answers. When the chatbot did not understand what the user was asking, instead of clarifying how to rephrase the input, it simply provided general guidance on how to return to the home menu. Additionally, some users said that the chatbot’s predefined answers sometimes did not match the guidance they expected.

I wanted to know how many items I needed to buy, so I searched for it, but all I got was “you can buy them at *** medical equipment store,” without any details.

[Female, 40s, caregiver, P06]

*The questions were okay, but at times, the way the chatbot guided the answers did not match our intent.*

[Female, 20s, caregiver, P42]

*When I first asked the chatbot about disinfection, it said, “I can’t answer that. Please go back to the beginning.*”

[Female, 60s, caregiver, P38]

#### User Experience Issues

Users encountered several challenges with the chatbot. Users often felt confused when they navigated specific information related to their current inquiries because all contents were stacked in a chat window without divisions by information depth. For example, some users mentioned that even after being informed by health care providers about the location of instructional videos, they still struggled to find them within the chatbot. After closing the chatbot, users experienced difficulty resuming conversations with it owing to challenges locating it among existing chats on the messenger platform. Additionally, the chatbot’s avatar was displayed consistently regardless of the response context, making it difficult for users to intuitively distinguish between the various contents displayed on the screen. Meanwhile, older individuals encountered difficulties with the chatbot’s carousel navigation, finding the swipe-based interface unfamiliar. They suggested implementing list-based items for easier access and improved usability.

*I wish the link to the video related to the question asked could be displayed at the top. It’s hard to find.*

[Male, 40s, caregiver, P39]

*I’m still young, so using internet devices isn’t too difficult for me. But with chatbots that require swiping sideways, both my mom and dad struggle with that. I can help them if I am with them, but I think it is generally difficult for older adults. It might be easier for them if all the content appeared as a list on one screen instead of having to swipe side to side.*

[Female, 30s, caregiver, P02]

*What do I need to do to enter the chatbot channel again?*

[Female, 20s, caregiver, P26]

*But I don’t think the image was significant. I barely noticed it because it was just an image of “O.”*

[Female, 30s, patient, P43]

#### Lack of Personalization

Users felt that the chatbot did not take into account individual variability in PICC-related issues, which reduced its usefulness in clearly addressing users’ concerns. For example, 1 user found that the chatbot’s predefined questions and answers were finite, as they did not address trivial or personal concerns. Additionally, users pointed out that there needs to be a feature allowing them to customize the length of the responses generated by the chatbot.

*I wish there were some educational content that addressed minor concerns. It felt awkward to call about something so trivial, like asking, “I don’t have a rash, but what should I do about this itchy area?” My parent said that they felt flustered and didn’t know what to do. In a situation like this, I think it would be helpful to have some guidance on what to do. I don’t think there was any educational content that covered these minor concerns.*

[Male, 40s, caregiver, P39]

*The chatbot provides very standardized questions and answers. But real life does not involve only the issues covered in those questions, right? There are other things I am curious about as well. New questions come up from time to time, and if I could leave a question and receive personalized feedback, like in a 1:1 Q&A, that would make a difference. Instead, I just tap a button, see the same standardized answer, and that is it. That is why it didn’t feel engaging or personal to me.*

[Female, 30s, caregiver, P02]

*To be honest, patients are not the ones doing research and studying. So I just want you to tell me, “Do it like this.” Even in my mother’s case, she has an impatient personality, and people are like, “So what do I need to do?”*

[Male, 40s, caregiver, P47]

*The patient mentioned having a fever, so I asked what to do in that case, but the answer wasn’t very detailed. I wish there had been a bit more detail.*

[Female, 20s, caregiver, P26]

## Discussion

### Principal Findings

The aim of this study was to evaluate the feasibility of a chatbot designed to support PICC self-management by examining chatbot use rate, usability, and qualitative satisfaction among patients with cancer and their caregivers, using data from survey, usage logs, and interview. Regarding chatbot adoption for PICC management, half of the participants used the chatbot during the study period. The most popular topic among chatbot users was PICC line management (eg, timing of dressing changes and purchasing dressing materials), followed by questions about adjusting to daily life, symptoms after PICC insertion, and heparinization. In terms of user experience, the 3 main benefits of the chatbot were information accessibility, effective guidance, and psychosocial support. Despite these promising findings, there were 3 key challenges related to the chatbot: conversational issues, user experience issues, and a lack of personalization.

In terms of chatbot adoption for PICC management, at least half of the participants used the chatbot during the study period. In previous studies, the adoption rates of health care chatbots ranged from 35% to 80% [30,31]. The heterogeneity in adoption rates across studies might depend on specific topics addressed by chatbots or the availability of alternative resources. For example, a study reported that 80% (n=48) of 60 patients with pancreatic cancer accessed the chatbot providing genetic information [30]. In another study examining a chatbot for chemotherapy care, 58% (n=20) of 34 patients with gastrointestinal cancer used the chatbot at least once [31]. Conversely, a chatbot for postoperative guidance after ureteroscopy was used by only 35% (n=7) of 20 patients [32]. Their lower activation rate may be attributed to the availability of multiple alternative information sources [32]. Compared with other studies, this study included both patients with cancer and their caregivers and provided nurse-led education for PICC management prior to offering the chatbot. These specific conditions might have contributed to participants experiencing a lower need to obtain PICC-related information via the chatbot. The primary motivation for chatbot utilization was related to individual needs for acquiring knowledge or information to prepare for or cope with PICC-related symptoms or issues. Notably, most nonusers reported experiencing mild or no PICC-related problems, diminishing their perceived need for the chatbot. Therefore, when assessing chatbot feasibility, it may be essential to consider whether potential users are experiencing the specific circumstances related to their perceived needs for health information.

The most popular topic among chatbot users was PICC line management (eg, timing of dressing changes and purchasing dressing materials), followed by questions about adjusting to daily life, symptoms after PICC insertion, and regular heparinization. A study on PICC management in nursing facilities reported communication gaps, such as uncertainty about the last dressing change date or PICC removal timing, even between hospitals and nursing facilities [33]. Another study on adults with a PICC at home found that showering was the most common challenge, followed by difficulties with exercising, sleeping, and dressing [34]. The chatbot in this study was designed and implemented based on patients’ information needs on PICC management in a real-world setting. The chatbot allowed users to access reliable information whenever they wanted. Although the questions and answers in the chatbot were prespecified, the topics the patients inquired about were comparable to the findings from previous studies. This suggests that chatbots could be feasible for providing informational support to patients with PICC lines. However, given the rule-based nature of chatbots, continuous expansion of the information the chatbot covers would be necessary to ensure sustainability.

In terms of the chatbot’s usability, users mentioned that it was an accessible channel for effective PICC self-management guidance. In this study, the chatbot was designed to provide practical tips or information regarding PICC self-management in a structured format with succinct and plain language. The implementation incorporated patient health literacy considerations, adhering to evidence-based recommendations for health care chatbot interfaces derived from previous studies [35,36]. Users particularly appreciated the convenience and rapid access to relevant information provided by chatbots, which may have enhanced their perceived usefulness in this study [17]. In addition, the chatbot was helpful for them to receive proper psychosocial support regarding PICC management. Patients with cancer, who face disease and treatment uncertainties, seek reliable information and justify their feelings through social support networks [37]. Moreover, PICC management is unfamiliar to most patients with cancer, requiring high competency in its long-term maintenance [38]. These features of patients with cancer can increase their anxiety regarding potential complications and coping strategies for cancer-related problems [38,39]. The chatbot in this study allowed users to access reliable information about PICC-related issues without temporal or spatial constraints, addressing unmet self-care needs and providing psychosocial support. During the COVID-19 pandemic, when hospital visits and communication with health care providers were often limited, the chatbot served as a clinically meaningful alternative by offering timely, trustworthy guidance. This support helped reduce patient anxiety and might have contributed to better treatment adherence. Furthermore, although the chatbot was designed to mimic human conversation, participants did not perceive its interaction as equivalent to real human dialogue. Future research should quantitatively evaluate these supportive functions to better understand their impact on patient outcomes.

Despite the promising findings, the chatbot encountered 3 main challenges: conversational issues, user experience issues, and a lack of personalization. A natural language understanding engine was integrated into the chatbot, but it occasionally failed to accurately interpret users’ intentions. The system was primarily optimized for button-click interactions rather than free-text interactions to facilitate an intuitive user experience. However, these rule-based, static responses seemed to diminish conversational naturalness and had limitations in capturing the various nuances of individual expressions for the same PICC-related issues. A systematic review found that rule-based health chatbots often limited user engagement owing to constrained input and predefined dialogues [19]. To resolve these unmet needs, incorporating large language models (LLMs), such as GPT or Claude, into rule-based systems would be necessary [40,41]. These models may provide better situational awareness and more human-like communication, which allow for personalized support in PICC self-management [41]. Given patients’ documented preference for direct clinician interaction, the strategic implementation of LLMs may be required for health care chatbots to approximate human-like communication efficacy [42]. Nevertheless, LLMs may generate plausible but inaccurate responses (“hallucinations”), necessitating rigorous reliability assessment and empirical validation prior to the clinical implementation of LLM-oriented health care chatbots [43]. Independent of preceding considerations, swipe-based navigation in the chatbot impeded user engagement, particularly among older users. Swipe-based navigation was difficult for them, and they often preferred icon-based menus to reduce cognitive load [44,45]. To assist user navigation, the implementation of subtle visual affordances indicating interface functionality and hierarchically structured list-based layouts is recommended [46].

### Limitations

First, although patients or their caregivers are required to attend PICC education, those who voluntarily consented to participate in this study may be more interested in using digital technologies or more motivated to self-manage. While this study did not collect demographic data of those who did not consent to participate, further understanding this in future research would provide more insight into scalable implementation. Second, user experiences were assessed after approximately 1 month of chatbot use. While PICCs are often maintained for 3-6 months, these findings primarily reflect the early phase of use rather than long-term engagement. Thus, the 1-month study period may not fully capture sustained engagement patterns. However, research has shown that many complications and difficulties tend to arise within the first few weeks following insertion, making this period particularly critical for patient education and support [47]. Hence, the data collection during this initial phase offers important insights into the most vulnerable period of PICC self-management. Nevertheless, future studies should incorporate longitudinal follow-up to the association between chatbot use and PICC-related complications, the sustainability of chatbot engagement, and its long-term effects on self-management. Third, the absence of a widely adopted, validated Korean-language instrument specifically designed for assessing chatbot usability limited comparability with existing literature. At the time this study was conducted, chatbot-specific usability instruments were not widely adopted; therefore, similar to prior studies, usability was assessed by adapting items from existing general usability questionnaires to reflect chatbot-specific characteristics [48,49]. Subsequent studies published after the completion of our research have begun to adopt chatbot-specific instruments, such as the Chatbot usability questionnaire; however, a validated Korean-language version or culturally adapted chatbot usability measure remains unavailable. Future research would therefore benefit from the development and validation of culturally appropriate, standardized Korean-language chatbot usability instruments to enable more rigorous and comparable assessments across studies. Finally, the small sample size and single-site design limit the generalizability of the findings and the ability to draw definitive conclusions. Future studies with larger and more diverse samples will be necessary to validate quantitative findings and support more robust statistical comparisons. Moreover, because participants in this study received structured onboarding and brief training, uptake in routine clinical practice where such support may not be systematically provided could be lower. This possibility further underscores the need for implementation strategies tailored to diverse clinical environments.

### Conclusions

In this study, the feasibility of a chatbot-based supportive care system for managing PICC lines in patients with cancer and their caregivers was explored. The chatbot had potential in enhancing information access, providing guidance, and offering psychosocial support. In particular, during situations such as the COVID-19 pandemic when patients were unable to visit hospitals or easily contact health care providers, the chatbot served as a valuable alternative by delivering reliable information and independently supporting the resolution of minor issues, thereby improving access to medical guidance. However, limitations in conversational quality and personalization were identified. Further quantitative research is warranted to strengthen the evidence base for the chatbot’s effectiveness. Incorporating LLMs may enhance future chatbot performance and user experience.

## Supplementary material

10.2196/81026 Multimedia Appendix 1 Other reasons for using the peripherally inserted central catheter (PICC) chatbot (n=28).

10.2196/81026 Multimedia Appendix 2 Other reasons for not using the peripherally inserted central catheter (PICC) chatbot (n=28).

10.2196/81026 Multimedia Appendix 3 Category of user inquiries through the peripherally inserted central catheter (PICC) chatbot (n=25; 347 observations).

10.2196/81026 Multimedia Appendix 4 Perceived benefits and unmet needs of using the peripherally inserted central catheter (PICC) chatbot (n=56).

## Abbreviations

GRAMMS: Good Reporting of a Mixed Methods Study
LLM: large language model
PICC: peripherally inserted central catheter
