# Supplementary material for: User Experiences of a Chatbot for Supporting the Self-Management of Peripherally Inserted Central Catheter for Chemotherapy: Mixed Methods Study
Source: JMIR Cancer. 2026 Feb 11;12:e81026. doi: 10.2196/81026 (PMC12893643; doi:10.2196/81026)
Supplement: Multimedia Appendix 1 [file cancer-v12-e81026-s001.docx]

| Domains | Items | Quotations |
| --- | --- | --- |
| **Symptoms occurring** | Abnormalities at the catheter insertion site | “There was a slight rash where the catheter was inserted, and there was some sudden bleeding, so I consulted the chatbot.” –Female, 20s, Caregiver, P42  “I was told to go to the emergency room if I felt heat where the PICC was inserted, but it wasn’t easy to get there right away, so I had to look at the chatbot one more time.” –Male, 60s, Patient, P54 |
| **Repetitive learning to acquire information** | Management of daily life | “Even after receiving training, I still felt confused when I went home. I consulted the chatbot because I was unsure whether I could use waterproof tape for a week or if I needed to continue disinfecting.” –Female, 50s, Patient, P23  “I looked into whether I could stretch or exercise after inserting the catheter.” –Female, 50s, Patient, P30 |
|  | Caring for catheter | “I was a bit confused about the amount of heparin injection I needed to administer during self-management.” –Female, 30s, Caregiver, P35 |
|  | Emergency room visit | “If the chatbot says you need to go to the emergency room, it’s right to go to the emergency room.” –Female, 20s, Caregiver, P42 |
| **Anxiety regarding medical services at local hospitals** | Psychological stability | “If you receive catheter management only at the hospital where the PICC was inserted, it is not a problem at all because you can trust and feel safe. But now I am going to a nursing hospital. The place where the procedure is performed and the place where it is managed are different. […] So now I need to know the details to ensure it’s being managed properly.” –Female, 50s, Patient, P15  “Given that we’re on Jeju Island, we’ve actually relied on chatbots more. Otherwise, we’d have to continue living with anxiety… so we decided to rely on a chatbot first.” –Female, 20s, Caregiver, P42  “I live in a rural area. It is not possible to go to the metropolitan area every time there is a problem in the local area. I had to figure it out somehow […], so I called the hospital where it was inserted, but the phone didn’t connect. As that didn’t work, I had no choice but to use a chatbot at home.” –Female, 60s, Patient, P09 |
