# Supplementary material for: User Experiences of a Chatbot for Supporting the Self-Management of Peripherally Inserted Central Catheter for Chemotherapy: Mixed Methods Study
Source: JMIR Cancer. 2026 Feb 11;12:e81026. doi: 10.2196/81026 (PMC12893643; doi:10.2196/81026)
Supplement: Multimedia Appendix 2 [file cancer-v12-e81026-s002.docx]

| Domains | Items | Quotation |
| --- | --- | --- |
| **Did not feel the need to use chatbots** | No issues encountered | “Well, there weren’t any issues significant enough for me to require using the chatbot.” –Female, 60s, Patient, P10 |
|  | Resolved by medical staff | “When I take a shower and my PICC falls out, I think, ‘If this catheter falls out, I’m in big trouble,’ so I go to the hospital first instead of the chatbot, without even realizing it. […] I think, ‘I need to meet the medical staff and get treatment quickly.’ So the idea of using a chatbot doesn’t even cross my mind. I just head straight to the hospital.” –Female, 40s, Patient, P13  “Since the home nurse starting coming, I didn’t need to use the chatbot. They took care of everything.” –Male, 70s, Patient, P08 |
|  | Utilized handouts received during training | “Even though using the chatbot would enhance my understanding of catheter management, I feel that the booklet sufficed for me. Even without consulting the chatbot, we were provided with comprehensive knowledge about maintenance, disinfection procedures, etc., solely from the booklet. […] We belong to a generation that finds it much easier to grasp information from booklets. The booklet was very intuitive and provided clear explanations.” –Female, 50s, Patient, P15 |
| **Chatbot use is unfamiliar and difficult** | Not sure how to use it | “If I need it, I tell my daughter to go in and look for it, and I’ve never used it. If I get stuck while using a chatbot, I get frustrated and no matter what I do, I can’t do anything, so I don’t do it well.” –Female, 60s, Patient, P33 |
|  | Not familiar with using chatbots | “I wasn’t familiar with chatbots, so I looked it up on the internet.” –Female, 30s, Patient, P18 |
| **Lack of trust in chatbots** | Anxiety about personal information leakage | “I avoid turning on the chatbot because I’m afraid they might ask me to input my resident registration number.” –Female, 60s, Patient, P12 |
|  | Lack of trust in information provided by chatbots | “Because it’s a chatbot, you can’t trust it 100% because the chatbot is not responsible. When I talk to a person, it seems like they clearly listen to what I have to say and speak professionally, but even though the chatbot talks to me, I don't trust it 100%.” –Male, 50s, Caregiver, P24 |
| **Low expectations for chatbots** | Not as expected | “To be honest, I don’t believe it will be very helpful.” –Female, 40s, Patient, P13 |
| **Instability of the chatbot usage environment** | Not in an internet environment | “Because my phone bill is cheap, the internet doesn’t work well. Therefore, if it doesn’t work well, I simply choose not to use it.” –Female, 60s, Patient, P12 |
