# Supplementary figures and images for: User Experiences of a Chatbot for Supporting the Self-Management of Peripherally Inserted Central Catheter for Chemotherapy: Mixed Methods Study
Source: JMIR Cancer. 2026 Feb 11;12:e81026. doi: 10.2196/81026 (PMC12893643; doi:10.2196/81026)

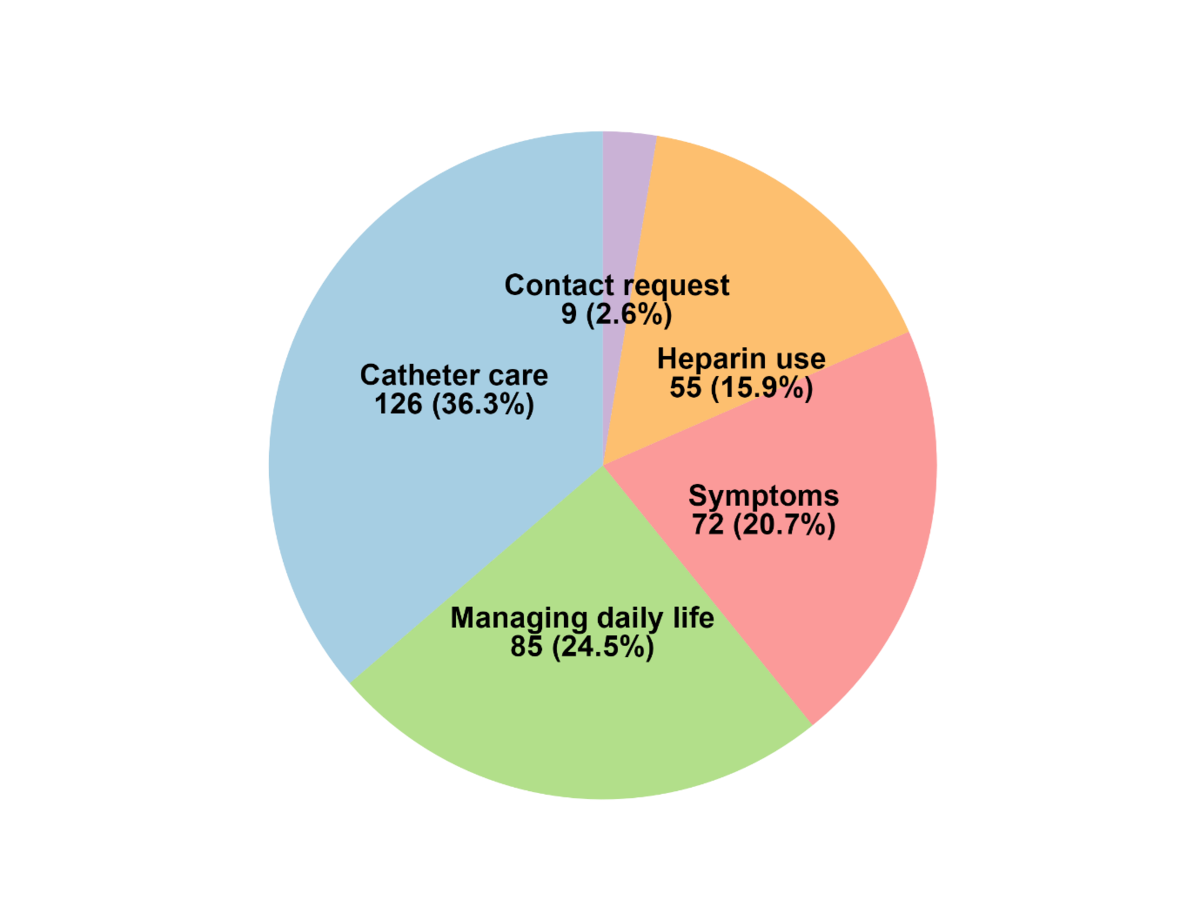

Supplement: Multimedia Appendix 3 [file cancer-v12-e81026-s003.png]
