# Supplementary material for: User Experiences of a Chatbot for Supporting the Self-Management of Peripherally Inserted Central Catheter for Chemotherapy: Mixed Methods Study
Source: JMIR Cancer. 2026 Feb 11;12:e81026. doi: 10.2196/81026 (PMC12893643; doi:10.2196/81026)
Supplement: Multimedia Appendix 4 [file cancer-v12-e81026-s004.docx]

| Classification | Domains | Items | Quotation |
| --- | --- | --- | --- |
| **Perceived Benefits** | Information Accessibility | Quick response available | “It was very helpful because I was able to receive an immediate response in real time. […] And when I needed information, I was able to simply inquire through KakaoTalk.” –Female, 60s, Patient, P56 |
|  |  | Easy to understand with short sentences | “I would like to say chatbots are understood more clearly and immediately because they provide information in a brief format, unlike paper materials. […] Anyway, chatbots are understood instantly” –Female, 40s, Caregiver, P06 |
|  | Effective Guidance | Confidence in self-management | “I checked whether it was correct to attach the tape after it had completely dried. […] I checked this and that and also double checked, ‘What am I doing wrong?’” –Female, 60s, Caregiver, P38  “When I looked at this here (chatbot), I thought I could figure out a solution.” –Male, 70s, Patient, P20 |
|  |  | Convenience of connecting with medical staff | “After doing some searching on the chatbot, I got in touch with the hospital again, the medical staff gave me a consultation, and I made an appointment for the next day. […] The best part was that it was directly connected to the hospital.” –Female, 40s, Caregiver, P06  “I think the video and phone number to contact the medical staff was also helpful. It was great that they told me to call this number if there were any issues.” –Female, 30s, Patient, P52 |
|  |  | Easy primary resolution | “I really liked the chatbot. It provided the primary solution, and I managed to resolve many issues through it. […] I solved a lot here (chatbot).” –Female, 40s, Caregiver, P06  “When I searched, the chatbot suggested that it might be a blood clot. So I quickly received primary treatment at the local hospital’s emergency room and then came back to the main hospital to have the PICC line removed.” –Female, 20s, Caregiver, P42 |
|  | Psychosocial Support | Emotional connection through familiar medical staff | “I was delighted because the video I watched here was exactly the same as the one I had seen during my training. I was happy to see the staff who taught me appear in the video.” –Female, 60s, Patient, P38 |
|  |  | Reassurance from having a support platform and proactive knowledge acquisition. | “If something went wrong, I needed to find a solution quickly, and I thought, ‘Who should I ask about this?’, but I really relied on this kind of thing. […] I depended on it and asked questions here (chatbot), so I felt much less anxious. Definitely.” –Female, 70s, Patient , P34  “It really helped me when I looked up things I was curious about, such as when to go to the emergency room, when to switch hospitals, and what to do if the tube falls out. Knowing this information ahead of time made me feel a little more at ease.” –Female, 60s, Caregiver, P19 |
|  |  | Reassurance from knowing symptoms are common | “I was looking into whether other people have these symptoms and if this is common or just something I am experiencing.” –Female, 60s, Caregiver, P19 |
|  |  | Reassurance provided through detailed information | “I think it helped reduce my anxiety a lot. I realized I had been more scared than I expected, but after getting information from the chatbot, I thought, ‘I don’t need to worry that much.’ For example, learning that catheter disinfection isn’t something to be that afraid of… and also finding out that I can go about daily life and even exercise without any major issues, that was really helpful.” –Female, 60s, Caregiver, P43 |
| **Unmet Needs** | Conversational Issues | Need to transition from a rule-based system to a conversational chatbot service | “I wanted to know how many items I needed to buy, so I searched for it but all I got was ‘you can buy them at *** medical equipment store,’ without any details.” –Female, 40s, Caregiver, P06  “The questions were okay, but at times, the way the chatbot guided the answers did not match our intent.” –Female, 20s, Caregiver, P42  “When I first asked the chatbot about disinfection, it said, ‘I can’t answer that. Please go back to the beginning.’” –Female, 60s, Caregiver, P38 |
|  | User Experience Issues | Need to increase font size | “Because we are at the age where presbyopia is beginning, it is not easy for us to read text, especially when there is a lot of it or when it’s crowded together. It can be quite uncomfortable for our eyes.” –Female, 50s, Patient, P15 |
|  |  | Content exposure order requires adjustment | “I wish the link to the video related to the question asked could be displayed at the top. It’s hard to find.” –Male, 40s, Caregiver, P39 |
|  |  | Requirement for tutorial feature | “I’m still young, so using internet devices isn’t too difficult for me. But with chatbots that require swiping sideways, both my mom and dad struggle with that. I can help them if I am with them, but I think it is generally difficult for older adults. It might be easier for them if all the content appeared as a list on one screen instead of having to swipe side to side.” –Female, 30s, Caregiver, P02  “What do I need to do to enter the chatbot channel again?” –Female, 20s, Caregiver, P26 |
|  |  | Need to use diverse images | “If the picture keeps showing the same circle and the same pose, I have to read the whole response. […] The picture has not changed. I think the pictures are saying the same thing. The answers are different, but the picture remains the same.” –Female, 40s, Caregiver, P06  “But I don’t think the image was significant. I barely noticed it because it was just an image of an ‘O.’” –Female, 30s, Patient, P43 |
|  | Lack of Personalization | Requirement for content diversification | “But now that I've watched it a few times, I’ve seen it all. I wish there was more content. […] After watching it a few times, I know everything, and I was curious about new content. I think it would be good if you could add something.” –Female, 40s, Caregiver, P06  “I wish there were some educational content that addressed minor concerns. It felt awkward to call about something so trivial, like asking, ‘I don’t have a rash, but what should I do about this itchy area?’ My parent said that they felt flustered and didn’t know what to do. In a situation like this, I think it would be helpful to have some guidance on what to do. I don’t think there was any educational content that covered these minor concerns.” –Male, 40s, Caregiver, P39 |
|  |  | Request a personalized response | “The chatbot provides very standardized questions and answers. But real life does not involve only the issues covered in those questions, right? There are other things I am curious about as well. New questions come up from time to time, and if I could leave a question and receive personalized feedback, like in a 1:1 Q&A, that would make a difference. Instead, I just tap a button, see the same standardized answer, and that is it. That is why it didn’t feel engaging or personal to me.” –Female, 30s, Caregiver, P02 |
|  |  | Requirement to tailor answer depth according to preference | “To be honest, patients are not the ones doing research and studying. So I just want you to tell me, ‘Do it like this.' Even in my mother’s case, she has an impatient personality, and people are like, ‘So what do I need to do?’” –Male, 40s, Caregiver, P47  “The patient mentioned having a fever, so I asked what to do in that case, but the answer wasn’t very detailed. I wish there had been a bit more detail.” –Female, 20s, Caregiver, P26 |
